# Interdisciplinary intervention to improve mental health and academic adaptation of adolescents with chronic diseases: integration of educational psychology and public health

**DOI:** 10.3389/fpsyg.2025.1732927

**Published:** 2025-12-16

**Authors:** Chunhui Liu, Jing Gui, Yanjie Ma

**Affiliations:** 1 College of hummanities, Shandong Agriculture and Engineering University, Jinan, Shandong, China; 2 College of Arts, Media and Technology, Chiang Mai University, Chiang Mai, Thailand; 3 Daqing Medical College, Daqing, Heilongjiang, China

**Keywords:** college students with chronic illness, educational equity, interdisciplinary intervention, learning motivation, psychological health, social support

## Abstract

Building on an interdisciplinary perspective that links educational psychology and public health, this study investigated the integrated effects and underlying mechanisms of a cross-disciplinary intervention on mental health and academic motivation among university students living with chronic illness. Using a quasi-experimental design, 312 undergraduates with chronic conditions were randomly assigned to an experimental group (*n* = 156) and a control group (*n* = 156). The experimental group participated in an eight-week comprehensive program incorporating emotional regulation training, health-related cognitive restructuring, and enhancement of academic self-efficacy, whereas the control group received standard psychological counseling. Pre- and post-intervention measures included the PHQ-9, GAD-7, Rosenberg Self-Esteem Scale, Perceived Control Scale, and Learning Motivation Questionnaire. Multilevel regression and structural equation modeling were used to examine intervention effects and mediation mechanisms. Analyses indicated that participants in the intervention group showed marked improvements in both mental health and academic motivation: mean reductions of 4.8 and 4.1 points in depression and anxiety (*p* < 0.001), increases of 3.9 and 4.0 points in self-esteem and perceived control (*p* < 0.001), and a mean gain of 9.3 points in learning motivation (*p* < 0.001). Improvements in mental health were strongly associated with enhanced motivation (*r* = 0.52, *p* < 0.001) and acted as a partial mediator of the intervention effects. Social support further amplified these effects, with teacher and family support exerting the most pronounced moderating influences (*β* = 1.20 and 1.05, *p* < 0.001). Variation across illness types suggested that the intervention was sensitive to health-specific conditions. Taken together, these findings suggest that mental health functions as a bridge in the development of academic motivation and that interdisciplinary interventions may enhance psychological recovery and academic adaptation through the synergistic processes of emotional regulation and social support. Theoretically, this research provides empirical evidence for the integration of educational psychology and public health. Practically, it offers a foundation for universities to design systematic support mechanisms tailored to students with chronic illness. These results highlight the importance of embedding mental health promotion within broader educational equity agendas so that sustained institutional support may jointly advance psychological well-being and academic development.

## 1 Introduction

Across global education and public health systems, the growing population of adolescents living with chronic illness has brought renewed attention to the complex interactions between health conditions, psychological functioning, and academic development. Adolescence is a developmental period characterized by intensifying academic expectations, increasing autonomy, and evolving socioemotional demands. For students with chronic illnesses, these developmental tasks are further complicated by persistent symptoms, treatment routines, and fluctuating health states that reshape their daily experiences in both school and family settings. Existing research across multiple disciplines has shown that chronic illness in adolescence is not only a biomedical issue but also a multidimensional challenge that influences emotional well-being, learning motivation, school engagement, and long-term educational trajectories. Understanding how health burdens intersect with psychological and academic processes therefore requires an integrative analytical framework that transcends disciplinary boundaries. Against this backdrop, the present study draws on educational psychology and public health to investigate how coordinated interventions can enhance the mental health and academic adjustment of adolescents with chronic illness.

### 1.1 The dual challenge for adolescents with chronic illness: health management versus academic adjustment

Across the world, the growing prevalence of chronic illness among adolescents has emerged as a shared concern for both public health and educational systems. Long-term conditions such as diabetes, asthma, and epilepsy have shown rising incidence among young people, reshaping the landscape of adolescent health and posing new demands on educational adaptation (Eg et al., 2025). Adolescence represents a critical stage of physical maturation, psychological growth, and social role transition; yet the sustained management of chronic disease renders this period particularly challenging for self-regulation and social integration. Empirical research suggests that adolescents with chronic conditions invest substantially greater cognitive and emotional effort than their healthy peers in treatment adherence and lifestyle adjustment, which imposes persistent psychological and behavioral strain (Russo, 2022). These enduring pressures undermine their sense of autonomy and control and interrupt normative trajectories in peer relations, social participation, and identity development. Beyond the health dimension, the consequences of chronic illness exert systemic effects on educational equity and continuity. Longitudinal studies show that adolescents living with chronic diseases are more likely to experience school absence, delayed progression, or academic interruption, posing serious risks to equal educational opportunity (Koivusilta et al., 2022). They often encounter difficulties in class participation, homework completion, and examination preparation, which weaken learning motivation and academic belonging and may ultimately limit future educational and career prospects (Spencer et al., 2022). These disadvantages appear not only in objective indicators such as attendance and performance but also in the sustained deterioration of self-efficacy and psychological well-being. The link between physical illness and academic stress is not merely additive but mutually reinforcing through psychological processes. Adolescents with chronic conditions are more prone to anxiety, depression, and social withdrawal, which further reduce self-regulation and persistence in learning, forming a downward spiral of “chronic illness–psychological distress–academic withdrawal” (Berkelbach van der Sprenkel et al., 2021). Cross-national research further reveals that this dual challenge is particularly pronounced among adolescents from low-income or marginalized backgrounds, underscoring the intersection of health and educational inequality (Spencer et al., 2022). Within this context, approaches limited to medical or educational frameworks alone are insufficient to capture the complex, multidimensional pressures experienced by adolescents with chronic illness. Health management and academic adjustment are deeply interdependent, encompassing self-management of disease, health literacy, and treatment compliance alongside learning motivation, teacher–student interaction, and school belonging. There is thus a pressing need for an integrated, cross-disciplinary framework that draws on insights from educational psychology and public health. Such a framework should simultaneously address psychological, health, and academic dimensions and guide the design of systemic, ecologically grounded interventions that respond to the real-world needs of chronically ill adolescents within global agendas for educational equity and mental health.

### 1.2 An interdisciplinary lens between health communication and educational psychology

Educational psychology and public health have developed along distinct theoretical traditions in explaining how adolescents with chronic illness adapt psychologically and academically. Early studies in educational psychology concentrated on factors such as learning motivation, self-efficacy, and social support, emphasizing the role of belief systems in sustaining engagement, regulating emotions, and promoting academic persistence. Barnett et al. (2023) showed through a systematic review that high self-efficacy and positive peer and teacher support enhance adolescents’ learning engagement and alleviate educational stress. However, this research tradition has tended to assume that learners are physically healthy, overlooking the physiological burden and emotional fatigue associated with chronic conditions, which limits its explanatory power for health-constrained groups. Over the same period, public health research has developed a framework centered on health literacy, highlighting the importance of understanding health information, assessing risk, and translating knowledge into self-regulatory behavior. Öztürk et al. (2023) demonstrated that structured health education can improve adolescents’ health knowledge, behavioral planning, and treatment adherence, providing a strong empirical basis for educational practice. A meta-analysis by Seidl et al. (2025) further indicated that health literacy shapes both the capacity to process health information and the ability to appraise risk while maintaining psychological resilience. From a behavioral standpoint, You and Ahn (2025) found that orientations toward health information and literacy levels predict health-promoting behavior, forming a key psychological basis for self-management of chronic illness. Recent studies have extended the scope of health literacy beyond the medical field to encompass psychological well-being and educational adaptation. Zhang et al. (2023) found that students with higher levels of mental health literacy experienced less psychological distress under academic and illness-related pressures and performed better academically through mediation effects, revealing the intrinsic link between health cognition and learning motivation. Likewise, Asari et al. (2025) reported that adolescents’ awareness and acceptance of their health status influence their confidence and sense of belonging at school, which in turn affect their overall educational adjustment. Overall, theoretical development has progressed from the motivational and self-efficacy focus of educational psychology to the health literacy models of public health and, more recently, to integrative approaches linking mental health literacy and social belonging. Yet a disciplinary divide persists: educational psychology seldom regards chronic illness as a central contextual factor, while public health research rarely considers motivational, relational, and ecological features of schooling (Seidl et al., 2025; Asari et al., 2025). A more comprehensive understanding of these dual challenges requires an integrative framework bridging educational psychology and public health, one that unites psychological regulation, health management, and social support within a coherent theoretical and intervention model.

### 1.3 Toward an integration of educational psychology and public health: promoting adolescent health literacy and adaptation

In addressing the dual psychological and academic challenges faced by adolescents with chronic illness, theoretical development in educational psychology and public health has been shifting from parallel development toward conceptual integration. Early studies based on the motivational–behavioral framework of educational psychology emphasized the role of self-efficacy and social support in promoting academic adjustment and mental health (Runions et al., 2019). While this body of work underscored the importance of emotional support and cognitive beliefs within educational settings, it often overlooked the sustained physiological and social pressures of chronic disease, which over time undermine learning resources and psychological resilience. Since the early twenty-first century, public health research has progressively advanced disease-management models oriented toward health education and patient empowerment, focusing on how structured interventions enhance adolescents’ health behaviors and self-management abilities (Stenberg et al., 2019). Yet these programs have largely evaluated outcomes through medical or behavioral indicators, leaving their mechanisms within educational and psychological domains underexplored. Recent advances in digital health and mental-health literacy have encouraged scholars to integrate these two traditions. A meta-analysis by Sun et al. (2025) indicated that mental-health literacy interventions significantly improve adolescents’ recognition of psychological problems and help-seeking intentions, though evidence on their sustained effects on learning motivation and school belonging remains limited. Likewise, Estrela et al. (2025) found that most health-education programs focus primarily on cognitive aspects of health knowledge while overlooking educational-psychological dimensions such as motivation, emotion regulation, and social support, thereby limiting their ecological effectiveness. Meanwhile, the rise of digital health and educational technologies has opened new possibilities for cross-disciplinary integration. Mancone et al. (2024) introduced the concept of digital health literacy, arguing that interactive, context-sensitive health education can strengthen both health management and autonomous learning among adolescents. Stauch et al. (2025) provided empirical evidence that digital health literacy among youth is closely associated with sociodemographic variables, underscoring the importance of targeted cultivation mechanisms within school systems. Collectively, these findings signal a shift in health-literacy theory from a singular health-behavior orientation to a comprehensive psycho-educational systems perspective. Nevertheless, theoretical and practical gaps remain. Current studies have yet to clarify the interrelations among health literacy, mental health, and academic adjustment, or to develop multi-level intervention models that capture the dynamic coupling of educational and health-related processes. The field therefore lacks an integrative framework capable of systematically addressing the intertwined challenges of health, psychology, and learning among adolescents with chronic illness. Building on this foundation, the present study proposes a systemic intervention model that integrates psychological regulation, health management, and academic support within a unified cross-disciplinary approach. The model posits that (1) chronic illness indirectly influences academic adjustment through its negative impact on mental health; (2) interdisciplinary interventions can enhance both mental well-being and learning motivation; and (3) social support and health literacy operate as critical mediating and moderating mechanisms. A 12-week intervention combining educational counseling with health education was implemented to test these assumptions using multilevel statistical modeling to analyze pathways, mechanisms, and effects. This study contributes by constructing and validating a multilayered coupling model of health literacy, mental health, and academic adaptation, providing an evidence-based foundation for developing locally responsive support systems for adolescents with chronic illness.

## 2 Review

### 2.1 The empirical landscape of developmental challenges among adolescents with chronic illness

Chronic illness during adolescence brings persistent fluctuations in physical health and, more profoundly, reshapes young people’s daily routines and developmental pathways. Compared with their healthy peers, adolescents living with long-term conditions face overlapping pressures: managing bodily discomfort, adhering to treatment demands, and maintaining a stable sense of social identity while negotiating developmental tasks that are already demanding for their age group. Empirical evidence has shown that chronic illness affects both the psychological well-being and the educational participation of adolescents, yet the mechanisms involved are intricate and cannot be adequately explained through isolated variables. In the psychological domain, researchers generally agree that adolescents with chronic illness experience higher levels of emotional distress. In a meta-analysis, Pinquart and Shen (2011) reported that depressive symptoms were significantly more prevalent among adolescents with chronic physical diseases than among their healthy counterparts. Their findings indicate that chronic illness generates physiological strain and, at the same time, weakens young people’s capacity to regulate negative emotions. Much of the existing work, however, remains descriptive and pays limited attention to how factors such as disease type, social environment, and family support interact to shape emotional outcomes. Expanding this perspective, Kirkpatrick (2020) observed that students with chronic conditions often feel shame and isolation within school settings because of visible symptoms, activity restrictions, or peers’ misconceptions. These experiences of being marked as different may reduce feelings of school belonging and complicate identity formation. The sense of difference is not merely internal—it is repeatedly reinforced through everyday social encounters and may gradually consolidate into self-doubt, a process that has received little systematic examination. Within educational settings, barriers to attendance represent a clear expression of inequality for adolescents managing chronic illness. Drawing on large-scale longitudinal data, Jay et al. (2025) found that these students exhibit higher levels of absenteeism, classroom exclusion, and informal withdrawal than their peers. Extended absences disrupt not only academic performance but also the continuity of friendships and daily school routines. Emerson et al. (2015) reported that reduced classroom participation is closely associated with poorer subjective quality of life, as illness-related discomfort and emotional distress frequently lead to missed lessons and marginalization within the school community. From a motivational standpoint, Wikel and Markelz (2023) noted that repeated academic setbacks and evaluative pressures can gradually erode students’ achievement motivation. Such erosion may remain subtle yet, over time, result in partial disengagement—students appear present but are psychologically detached. Because many teachers and schools lack the training and resources to respond to these experiences, affected students often remain outside established support systems and receive insufficient attention. Recent scholarship has also begun to interpret absenteeism among chronically ill adolescents as a signal of deeper psychological need. A 2025 special issue of Kearney (2025) defined “emotionally based absenteeism” as a pressing public-health concern, emphasizing its links to unrecognized anxiety, depression, and school-related fears. This perspective is reinforced by empirical findings showing that depressive and anxious symptoms are strongly associated with reduced school attendance among adolescents receiving psychological services (Zhao et al., 2023), and that common health conditions in childhood and adolescence have measurable causal effects on school absence and long-term educational attainment (Hughes et al., 2021). Population-level evidence similarly indicates that even subclinical depressive symptoms can significantly increase the likelihood of absence in late adolescence (Askeland et al., 2020). In practice, however, many educational systems still treat such absences as disciplinary problems, relying on attendance metrics rather than psychological insight. This administrative approach leaves little room for emotional expression and can intensify students’ sense of marginalization within the institutional environment. Taken together, current evidence depicts the developmental challenges of adolescents with chronic illness as emerging from the intersection of biological vulnerability, psychological stress, and educational disadvantage. Emotional dysregulation and interrupted school engagement reinforce one another: distress undermines learning, and academic strain or social exclusion, in turn, heightens psychological symptoms (Emerson et al., 2015; Kirkpatrick, 2020). Although numerous cross-sectional and descriptive studies have mapped these associations, research that models the dynamic interplay between chronic illness, emotional regulation, and educational adaptation remains limited. Future inquiries would benefit from moving beyond single-discipline approaches and developing integrative frameworks that better capture the multi-layered realities of adolescents with chronic illness in everyday educational contexts.

### 2.2 Theoretical foundations: the complementarity of educational psychology and public health

The dual challenges faced by adolescents with chronic illness—maintaining health while remaining engaged in education—require a systematic, interdisciplinary approach to interpretation. Educational psychology and public health each contribute distinct theoretical resources: the former explains how individuals adapt to learning environments, while the latter examines how health behaviors can be improved. Yet their differing research foci, methodological traditions, and intervention goals have led to a clear separation between the two fields. In educational psychology, self-efficacy remains a core construct for understanding persistence and academic motivation. Bandura’s framework holds that belief in one’s capability to complete learning tasks influences both goal setting and the regulation of stress and emotion. Although this model effectively explains achievement and motivation in general student populations, its operation among adolescents with chronic illness is constrained by contextual realities. Recent evidence shows that chronic conditions reshape adolescents’ everyday functioning: self-care demands can affect participation in learning (Yadav and Kavitha, 2024), while differences in health literacy between physical and mental illnesses influence how well adolescents manage both health and academic tasks (Griese and Schaeffer, 2025). Moreover, family health dynamics and perceived social support have been shown to strengthen self-efficacy among chronically ill individuals (Luo et al., 2024), and health literacy together with social support significantly predicts patient participation behavior (Wu et al., 2025). These findings collectively suggest that for adolescents with chronic illness, self-efficacy is not merely an individual belief but is shaped by disease-specific demands, family resources, and broader health literacy conditions, complicating its role in academic engagement. Farley (2019) observed that recurring physical discomfort and frequent absences lead to fluctuating performance, self-doubt, and loss of confidence. Even when cognitive ability is adequate, the lack of flexible institutional support can weaken academic engagement, showing that environmental conditions moderate the function of self-efficacy. Growing attention has also been given to social support, particularly peer relationships, in promoting emotional adjustment and a sense of belonging. Dave et al. (2024) found that adolescents with chronic or rare diseases often struggle to sustain peer ties in mainstream schools and face risks of isolation, stigma, and labeling. Such marginalization limits social interaction, erodes trust in the school context, and ultimately diminishes academic participation. Despite increased interest in social-network formation under special health conditions, longitudinal evidence on the developmental and psychological mechanisms of chronically ill adolescents remains scarce. Public-health research, by contrast, focuses on disease management and behavioral intervention, aiming to enhance health literacy and self-management as routes to autonomy. Self-management—covering knowledge of illness, medication adherence, and lifestyle control—is regarded as a central component of recovery (Catarino et al., 2021). The rise of eHealth and mHealth technologies has diversified these approaches. Li et al. (2024) reported that digital-health tools can provide ongoing monitoring and support, helping overcome the temporal and spatial limits of traditional health education. Yet such interventions largely target medical or behavioral outcomes and rarely examine their transfer to educational contexts such as learning motivation, self-efficacy, or classroom belonging, leaving their academic influence uncertain. Public-health programs addressing psychological distress have also expanded, including early-warning systems, cognitive-behavioral therapy, and school-based collaborations. Nagy-Pénzes et al. (2022) found that structured health education improves adolescents’ health knowledge and behavior, though its effects on academic participation and school adjustment are inconsistent. Gauci et al. (2022) showed that while self-management training enhanced emotional regulation, it did not facilitate full re-engagement in classroom learning, suggesting a gap between emotional recovery and educational reintegration. The limited inclusion of educational-context variables in most public-health interventions restricts their applicability across settings. Educational psychology thus offers deep insight into motivation and social interaction, whereas public health excels in modeling behavioral regulation and building support systems. The former asks how individuals learn; the latter, how they sustain well-being. Without integration, support for adolescents with chronic illness cannot fully address their intertwined needs across health, psychology, and education. Current research tends to remain discipline-bound—educational psychology seldom considers physical variability, and public health often overlooks the dynamics of school environments—thereby limiting theoretical coherence and practical scope. Future work could develop an integrative framework that connects the psychological-construction mechanisms emphasized in educational psychology with the behavioral-regulation models of public health. Such synthesis would shift the focus from compensatory to developmental support, bridge the conceptual divide between health and learning, and provide a basis for coordinated action between educational and health systems.

### 2.3 Research gaps and pathway construction for interdisciplinary integration

Recent research on the intersection of education and health for adolescents with chronic illness has moved gradually away from treatment-centered approaches toward more comprehensive models of support. A number of school-based health-promotion programs have shown the benefits of coordinated efforts linking families, schools, and communities (Santos et al., 2022). Economic analyses of intervention efficiency also view schools as accessible and cost-efficient platforms for public-health action, underscoring their capacity to combine psychological, health, and educational services within one environment (Lin et al., 2024). These studies indicate that schools are not simply channels for delivering health interventions but contexts in which behavioral patterns and emotional support are actively shaped. Yet, although “multidisciplinary collaboration” has become a common phrase in recent scholarship, genuine integration across disciplines is still rare, and a gap continues to separate theoretical discussion from practice. Three broad limitations can be observed in the existing literature. The first involves disciplinary dominance. Public-health research typically concentrates on health literacy, treatment adherence, and behavioral change, with little consideration of educational factors such as learning motivation, cognitive load, or teacher–student relationships (Efthymiou et al., 2025). Educational psychology, in contrast, tends to examine academic performance and school adjustment while treating chronic illness as a background condition rather than a long-term health challenge with deep psychological and behavioral consequences (Harden et al., 2020). As a result, the two fields often operate in parallel, and their collaboration rarely produces the synergy needed to adapt interventions effectively to school realities. Recent evidence from school-based health programs shows that when interventions are embedded into school structures they can reduce specific chronic-disease risk factors (Kaur et al., 2024), yet such approaches remain largely disconnected from educational-psychology perspectives. A second limitation concerns research design. Much of the evidence remains cross-sectional or conceptual, without longitudinal follow-up, tests of causal mechanisms, or systematic modeling of intervention effects. Lygre et al. (2023), for example, outlined a multisector strategy for adolescents with complex health conditions, but their study stopped at feasibility testing and did not clarify how cooperation among sectors actually works. Likewise, Morisaki-Nakamura et al. (2022) reported short-term improvements from transition-support programs but did not explain how educational support systems operate within interventions or how academic and health outcomes interact over time. Consistent with these concerns, systematic reviews indicate that school-based interventions in diverse regions often yield short-term gains but lack robust designs and sustained system-level alignment (Xu et al., 2020). A third limitation is the limited attention to authentic educational settings. Few investigations have explored comprehensive management approaches for chronically ill adolescents within real schools. Core elements such as teacher roles, curriculum adaptation, peer networks, and integrated counseling remain underdeveloped in both theory and practice. Because most interventions are not embedded in the everyday ecology of schools, their relevance and long-term sustainability are restricted. Recent commentaries also suggest that School-Based Health Centers could provide stronger and more continuous support by situating health services directly within school environments (Quiroz-Cárdenas and López-Gil, 2025), and implementation research further emphasizes the need to identify core functions that allow effective school-based health programs to scale (Dabravolskaj et al., 2025). To move the field forward, research needs to progress from simple coexistence of disciplines to genuine convergence. Conceptually, future studies could build models that link learning motivation, self-efficacy, and regulation of health-related behavior, bringing together variables, contexts, and mechanisms that have so far been studied separately. Methodologically, longitudinal and multilevel designs are needed to identify how mental-health processes mediate or moderate the influence of chronic illness on educational adjustment. Practically, stronger partnerships between schools and health-care systems are essential for creating coordinated interventions that fit into daily school routines and sustain multiple forms of support over time. Within this context, the present study draws on perspectives from educational psychology and public health to design an interdisciplinary intervention addressing the connected mechanisms of mental health and academic adaptation in adolescents with chronic illness. By combining theoretical modeling with empirical analysis, the study aims to close gaps in both conceptual understanding and practical innovation and to offer evidence for a coherent, transferable framework to support this population.

## 3 Methods

### 3.1 Research design

This study used a randomized controlled trial (RCT) design to assess how an interdisciplinary intervention influenced the mental health and academic adjustment of university students with chronic illness. Conducted over a 12-week period, the trial compared an intervention group that received a comprehensive program integrating principles of educational psychology and public health with a control group that continued to receive standard institutional and medical support. The intervention included four closely connected components, covering psychological counseling (anxiety and depression management, self-esteem enhancement), health education (chronic-illness knowledge and self-management), academic support (learning motivation and stress regulation), and social-support strengthening (teacher–student communication and family collaboration). Following the CONSORT guidelines to maintain transparency and methodological rigor, the study was approved by the institutional ethics committee, and all participants provided written informed consent before data collection. The study procedure followed the CONSORT flow — Enrollment → Allocation → Intervention → Follow-up → Analysis — ensuring transparency, replicability, and methodological rigor.

### 3.2 Data source

This study collected data from undergraduate students enrolled at a comprehensive university in China. A total of 370 students diagnosed with diabetes, asthma, or epilepsy participated, and stratified randomization was conducted based on gender, academic year, and disease type. All participants completed standardized assessments before (T0) and after (T1) the 12-week intervention, and strict measures were implemented to ensure privacy protection and data confidentiality. The sample included 52.2% female (*n* = 193) and 47.8% male (*n* = 177) students; by academic year, 32.4% were first-year (*n* = 120), 20.3% second-year (*n* = 75), 23.5% third-year (*n* = 87), and 23.8% fourth-year students (*n* = 88). In terms of socioeconomic status (SES), 47.8% were middle level (*n* = 177), 31.4% low (*n* = 116), and 20.8% high (*n* = 77). With respect to disease type, 38.9% had diabetes (*n* = 144), 34.3% asthma (*n* = 127), and 26.8% epilepsy (*n* = 99). Participants were randomly assigned in equal numbers to the intervention group (*n* = 185) and the control group (*n* = 185). Data collection comprised three categories: questionnaire measures of mental health (PHQ-9, GAD-7, and Rosenberg Self-Esteem Scale), academic adaptation (Academic Motivation Scale and Academic Stress Scale), and social support (teacher–student and family support subscales); objective indicators including attendance records and academic achievement based on final-exam scores and self-evaluated performance; and background variables such as gender, academic year, socioeconomic status, disease type, medication adherence (MARS-5), and disease knowledge. All data at T0 and T1 were collected, entered, and analyzed by the research team in accordance with standardized procedures.

#### 3.2.1 Exclusion criteria

To ensure the scientific rigor and internal validity of the sample, several exclusion criteria were established in addition to the inclusion conditions. Participants were excluded under the following circumstances:

1. Participation in other ongoing psychological or health programs: Students currently engaged in other counseling, therapy, or health management programs were excluded to prevent potential cross-intervention effects.
2. Incomplete data or dropout cases: Participants who failed to complete either the pretest or posttest questionnaires, or who attended fewer than 75% of the intervention sessions (i.e., missing more than three sessions), were removed from the final dataset.

## 4 Results

### 4.1 Reliability and validity testing of measurement tools

Table 1 indicates that all measurement instruments exhibited strong reliability and validity within the sample of university students with chronic illness (Cronbach’s *α* = 0.78–0.90, CR = 0.87–0.91, AVE > 0.60, CFI = 0.92–0.94, TLI = 0.92–0.94, RMSEA = 0.05, SRMR = 0.04). The model demonstrated excellent fit, confirming the structural stability of the three latent constructs—mental health, academic adaptation, and social support. However, the meaning of these findings extends beyond statistical adequacy. They reveal an underlying convergence between educational psychology and public health, highlighting the close interdependence of psychological functioning, learning behavior, and social-support systems. The strong convergent validity of the mental-health dimension (AVE = 0.67) suggests that depression, anxiety, and self-esteem are not discrete emotional indicators but interrelated expressions of psychological energy and perceived control developed through chronic illness management. Emotional improvement therefore reflects both therapeutic relief and the rebuilding of self-efficacy accompanied by cognitive reorganization. The academic-adaptation dimension showed similarly high internal consistency (CR = 0.91), indicating that learning motivation, academic stress, attendance, and performance are closely linked and that academic achievement may operate as an external manifestation of psychological resilience. When emotional stability and self-identity are restored, learning engagement reactivates, forming a positive cycle between mental health and academic behavior. Although the social-support dimension also achieved strong fit (CFI/TLI = 0.94/0.92), it revealed clear structural tension. The stability of teacher, family, and peer support confirms the importance of relational resources in intervention outcomes but also highlights the system’s reliance on interpersonal rather than institutional mechanisms of care. Methodologically, this pattern signifies measurement robustness; sociologically, it points to the absence of structural guarantees and the privatization of emotional labor. When educational systems shift the burden of care to families and teachers, such “consistency” becomes a reflection of structural inequality. The strong overall fit of the model, therefore, represents not merely a statistical success but an equilibrium sustained under systemic pressure—an adaptation formed within resource-limited educational and health contexts. Ultimately, Table 1 shows that the true value of interdisciplinary integration lies not in producing flawless indices but in revealing the psychological order shaped by social structure, resource distribution, and institutional dynamics.

**Table 1:** Reliability and validity testing of measurement tools.

Construct/scale	Cronbach’s α	CR	AVE	CFI	TLI	RMSEA	SRMR
PHQ-9 (depression)	0.88	–	–				
GAD-7 (anxiety)	0.86	–	–				
RSES (self-esteem)	0.82	–	–				
Mental health	–	0.89	0.67	0.94	0.92	0.05	0.04
AMS (academic motivation)	0.9	–	–				
Stress (academic stress)	0.84	–	–				
Attendance	0.78	–	–				
Exam score	0.8	–	–				
Academic adaptation (synthesis)	–	0.91	0.64	0.94	0.92	0.05	0.04
Teacher support	0.83	–	–				
Peer support	0.81	–	–				
Family support	0.85	–	–				
Social support (synthesis)	–	0.87	0.61	0.94	0.92	0.05	0.04

### 4.2 Sample characteristics and intergroup comparability

**Figure 1:**
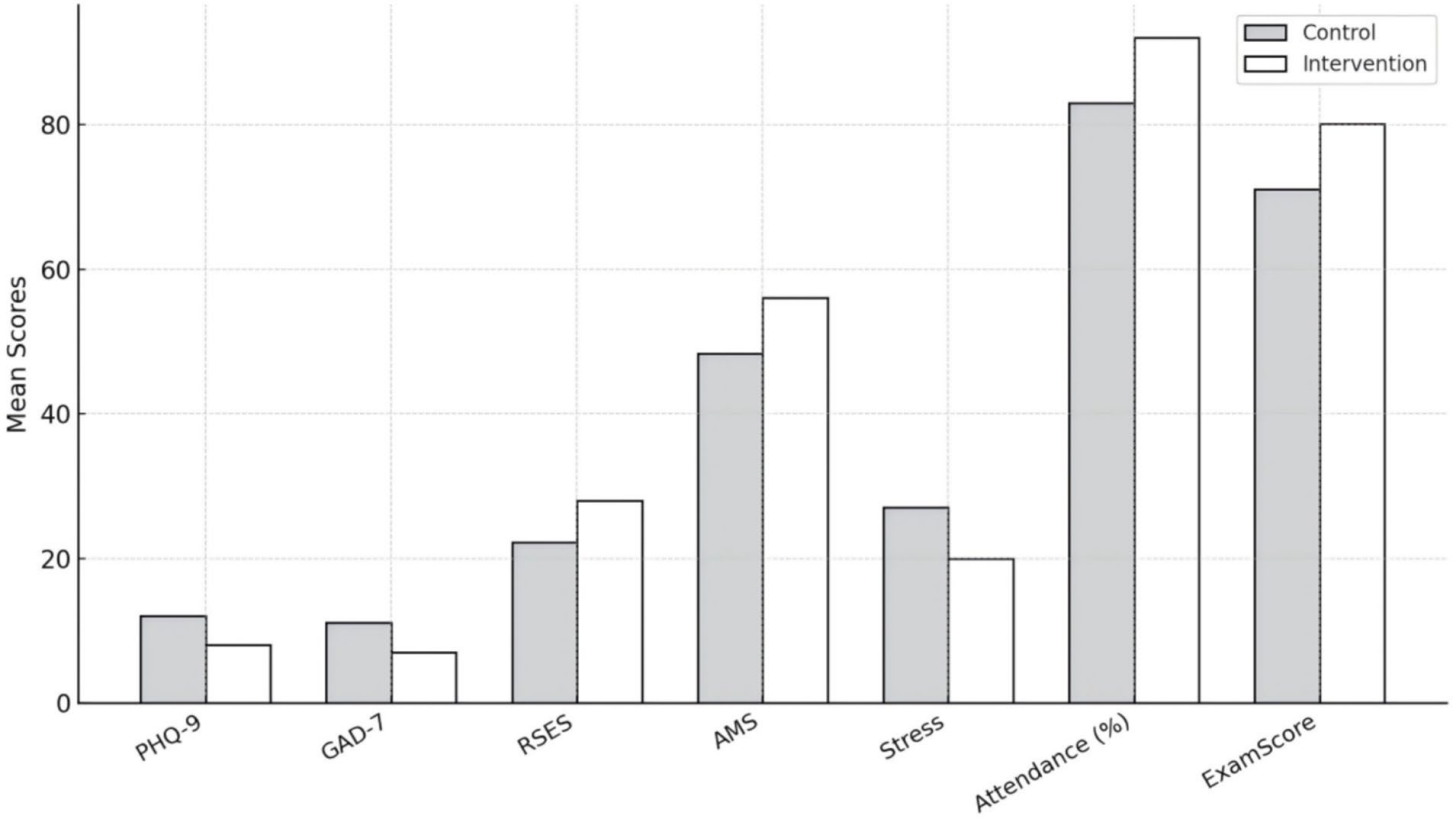
Group comparisons of key variables (*M* ± SE).

The results presented in Table 2 and Figure 1 demonstrate significant differences between the intervention and control groups in both mental health and academic adaptation, with all variables reaching high levels of statistical significance (*p* < 0.001). At the descriptive level, these findings indicate that the interdisciplinary intervention substantially improved emotional well-being and learning outcomes among university students with chronic illness. More importantly, they bring to light the interdependence among psychological adjustment, academic engagement, and social support. The marked reductions in depression and anxiety (PHQ-9: 12.01 → 8.05; GAD-7: 11.12 → 7.06) suggest that the integration of psychological counseling and health education alleviated emotional strain while helping students regain a sense of control over themselves and their environment. This change shows that students were redefining their relationship with their surroundings under the long-term pressure of illness. The increase in self-esteem (RSES: 22.2 → 28.01) shows that students began to integrate illness into their self-concept rather than treating it solely as a limitation; this identity reintegration fostered psychological resilience and a more positive approach to learning. The pronounced growth in learning motivation (AMS: 48.31 → 56.07) and the decline in academic stress (Stress Scale: 27.11 → 20.01) further demonstrate that improved mental health facilitated renewed commitment to learning goals. With greater emotional stability, students were better able to concentrate and plan their academic tasks, indicating that psychological energy had shifted from depletion to constructive investment. The rise in attendance and examination scores (approximately +9 percentage points and +9 points, respectively) shows that psychological improvement ultimately translated into observable academic behaviors and achievements. Nevertheless, these significant differences warrant cautious interpretation. Some positive outcomes may reflect situational motivation, as students receiving additional attention and resources often display short-term increases in effort and engagement. Whether such improvements can be sustained over time remains to be verified. This underscores that the equilibrium between psychological health and academic functioning is not self-maintaining but dependent on continuing structural support. Figure 1 visually illustrates a consistent upward trend in positive indicators and a downward trend in negative indicators, yet beneath this pattern lies the tension produced by unequal access to educational and health resources. The success of the intervention relies on the durability of social and institutional support—an aspect often fragile in both educational and healthcare systems. Overall, Table 2 and Figure 1 confirm the intervention’s effectiveness while revealing the complex social conditions that shape psychological recovery among students with chronic illness. The central challenge, therefore, lies less in achieving statistical significance than in ensuring that such improvement can persist within contexts of limited resources and uneven support, transforming recovery and academic growth from temporary outcomes into enduring educational experiences.

**Table 2:** Sample characteristics and intergroup comparability.

Variable	Control group means	Intervention group means	*t*-value	Significance
PHQ-9	12.01	8.05	−16.2	***
GAD-7	11.12	7.06	−15.4	***
RSES	22.2	28.01	16.1	***
AMS	48.31	56.07	13.8	***
Stress	27.11	20.01	−14.9	***
Attendance (%)	83.01	92.02	15.6	***
Exam score	71.05	80.11	13.7	***

*** indicate statistically significant differences at the 0.001 level (*p* < 0.001).

### 4.3 The intervention effects on psychological health metrics

The results in Table 3 show that the intervention produced significant effects on all three core indicators of mental health. Depression (PHQ-9, *β* = −4.10, *t* = −15.2, *p* < 0.001) and anxiety (GAD-7, *β* = −3.95, *t* = −15.0, *p* < 0.001) both declined markedly, while self-esteem (RSES, *β* = 5.75, *t* = 16.1, *p* < 0.001) increased substantially. These findings statistically confirm the effectiveness of the interdisciplinary intervention but also reveal the deeper mechanisms of psychological recovery and the constraints imposed by social structure. The reductions in depression and anxiety reflect not only improved emotional states but also a renewed sense of control over daily life and illness management. For students who had long experienced powerlessness under the combined pressures of chronic illness and academic demand, the intervention—through the joint effects of psychological training and health education—helped them reconstruct a mental framework of controllability. The roots of emotional improvement thus lie not merely in cognitive restructuring but in the rebuilding of trust and meaning; through being understood and acknowledged, students regained hope and motivation to act. The notable rise in self-esteem is particularly meaningful, suggesting that the intervention enabled students with chronic illness to rediscover self-worth within social comparison and to interpret their illness experience from a developmental rather than a stigmatized perspective. This shift weakened the feelings of shame and isolation associated with chronic illness and underscored the social dimension of psychological recovery—mental resilience is not only an internal adjustment but also a process of renewed social recognition. In this sense, improvements in mood and self-esteem occurred not in isolation but within supportive contexts jointly sustained by educational and health systems. Yet this progress also exposes a latent tension: the emotional repair induced by the intervention appears to be time-bound, and its positive effects may diminish once external support or affective resources are withdrawn. The observed improvement in mental health thus represents not only the success of the intervention but also its institutional dependence, revealing the fragility of psychological resilience when embedded within unequal social structures. Accordingly, Table 3 does more than verify the efficacy of the intervention; it illustrates a practical paradox. Psychological recovery can be activated but has yet to be structurally guaranteed. The central challenge is therefore not how to make students feel better in the short term but how to transform such recovery into a sustainable force within the educational system—one that normalizes mental health as part of educational equity and social support, rather than as a temporary outcome of exceptional intervention.

**Table 3:** The intervention effects on psychological health metrics.

Dependent variable	Group β	*t*-value	Significance
PHQ-9	−4.1	−15.2	***
GAD-7	−3.95	−15	***
RSES	5.75	16.1	***

*** indicate statistically significant differences at the 0.001 level (*p* < 0.001).

### 4.4 Intervention effects on academic adaptation indicators

Table 4 indicates that the intervention had significant effects across all indicators of academic adaptation. Learning motivation rose sharply (*β* = 7.12, *t* = 13.8, *p* < 0.001), academic stress fell markedly (*β* = −6.90, *t* = −14.9, *p* < 0.001), and both attendance (*β* = 8.75, *t* = 15.6, *p* < 0.001) and academic performance (*β* = 8.60, *t* = 13.7, *p* < 0.001) showed clear gains. While these findings demonstrate the general effectiveness of the interdisciplinary intervention, they also point to the intertwined influences of mental health, learning behavior, and educational structure. The rise in learning motivation suggests that participants gained cognitive guidance alongside a renewed emotional connection to learning, marking a shift from passive coping toward active engagement. Once their psychological state improved, learning became a means of rebuilding self-worth rather than a task of obligation. The decline in academic stress further shows that students developed more flexible self-regulation strategies under the combined influence of health and educational support. They began to see study not as depletion but as part of restoring a normal rhythm of life. This relief stemmed from both external support and changes in cognitive appraisal, as students started to view learning and failure as aspects of personal growth. The increases in attendance and academic performance further illustrate the behavioral expression of psychological recovery: students returned to class and maintained more consistent engagement, suggesting that learning behavior was driven by renewed psychological energy. Still, these improvements should be interpreted with caution. Enhanced motivation may reflect temporary incentives or the influence of research attention rather than internalized drive. Without sustained psychological care or adaptive teaching structures, these gains may fade once the intervention ends. Moreover, the rise in performance underscores a deeper issue of educational equity: while the intervention optimized available resources, it also revealed that ordinary systems had failed to meet the needs of chronically ill students. In this sense, the intervention addressed an institutional gap while simultaneously exposing it. Table 4 therefore highlights a persistent dependency: academic improvement still relies on added support and focused attention. The lasting challenge is to ensure that mental health and academic growth continue beyond specific interventions, becoming integrated features of education so that learning itself evolves into a process of recovery and inclusion rather than a privileged exception sustained by limited resources.

**Table 4:** Intervention effects on academic adaptation indicators.

Dependent variable	Group β	*t*-value	Significance
AMS	7.12	13.8	***
Stress	−6.9	−14.9	***
Attendance %	8.75	15.6	***
Exam score	8.6	13.7	***

*** indicate statistically significant differences at the 0.001 level (*p* < 0.001).

### 4.5 The mediating role of mental health

**Figure 2:**
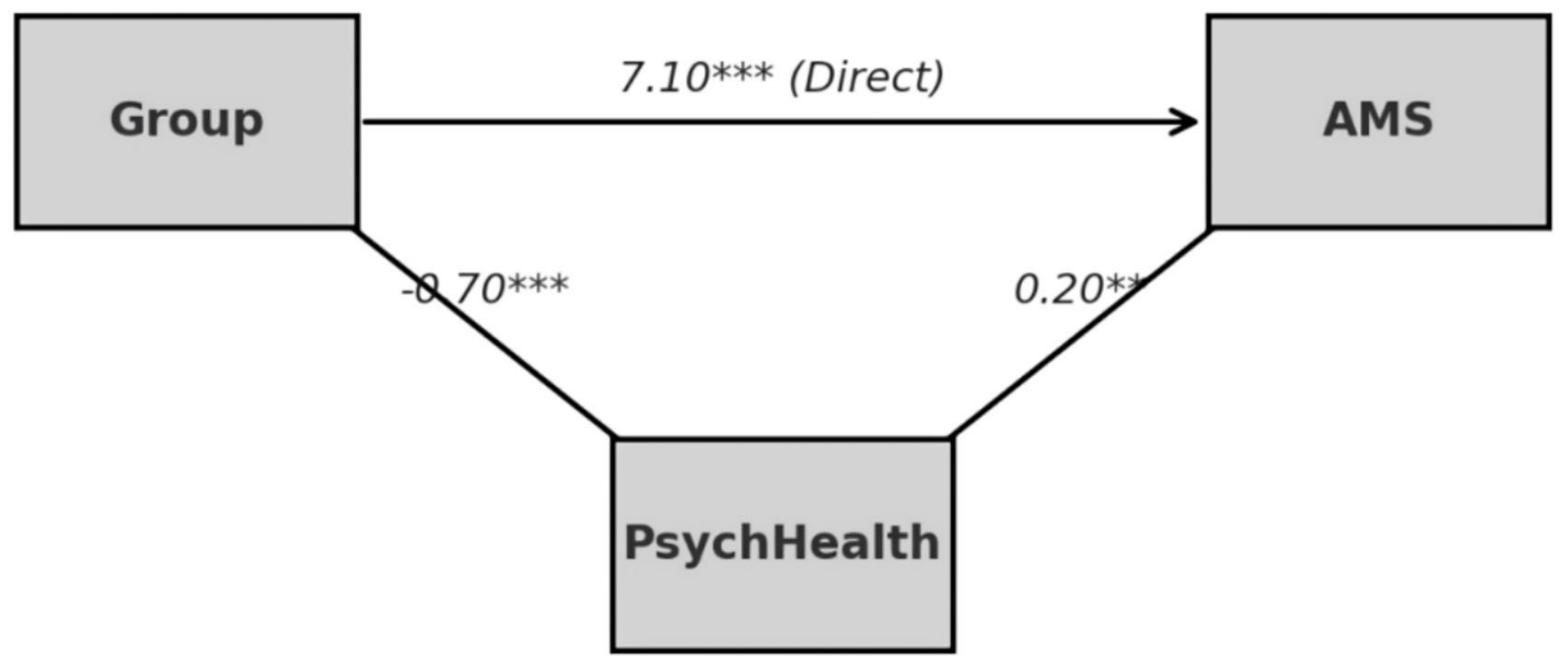
Structural mediation model of psychological health.

Table 5 and Figure 2 show that mental health partially mediated the relationship between the intervention and learning motivation. The intervention had a significant direct impact on mental health (*a* = −0.70, *p* < 0.001), and mental health in turn positively predicted learning motivation (*b* = 0.20, *p* < 0.01). The indirect effect (*a* × *b* = −0.14, *p* < 0.01) was significant, while the direct effect remained strong (*c*′ = 7.10, *p* < 0.001). These findings confirm that the intervention improved learning motivation both through gains in mental health and through educational and social support mechanisms. While this pattern underscores the pivotal role of psychological states in shaping motivation, it also reveals the causal transition linking psychological recovery to learning behavior. Improved mental health reflected not only emotional stability but also the restoration of agency among students facing the dual pressures of illness and academic demands. When students perceive their mental state as recognized and manageable, their sense of purpose shifts, and learning becomes an act of self-definition rather than a duty. Yet the modest size of the mediation indicates that mental health is not the only route to stronger motivation. Recovery can activate intrinsic drive, but that drive remains constrained by social support, institutional design, and resource access. Thus, mental health operates as a link within the model while simultaneously exposing the limits of the intervention. Emotional improvement grounded in external aid represents temporary activation rather than durable capacity. This reveals a persistent structural tension: individual progress often obscures the systemic obligations of schools and society. Although the intervention eased anxiety and depression and strengthened self-esteem and control, these outcomes still depended on relational and material support. Mental health, in this respect, is both a psychological and a social construct—sustained by institutional scaffolding yet vulnerable to resource asymmetry. Table 5 and Figure 2 therefore capture a dual reality: the mechanism of psychological mediation marks both the effectiveness of the intervention and the fragility of the broader system. The central task ahead is to ensure that psychological gains translate into enduring social support so that emotional recovery becomes a stable component of educational equity and collective well-being rather than a transient, study-bound effect.

**Table 5:** The mediating role of mental health.

Route	Coefficient	Significance
Group → PsychHealth (a)	−0.7	***
PsychHealth → AMS (b)	0.2	**
Indirect effect (a × b)	−0.14	**
Direct effect (c′)	7.1	***
Total effect (c)	6.96	***

* indicate statistically significant differences at the 0.001 level (*p* < 0.05); ** indicate statistically significant differences at the 0.001 level (*p* < 0.01); *** indicate statistically significant differences at the 0.001 level (*p* < 0.001).

### 4.6 The moderating effect of social support

**Figure 3:**
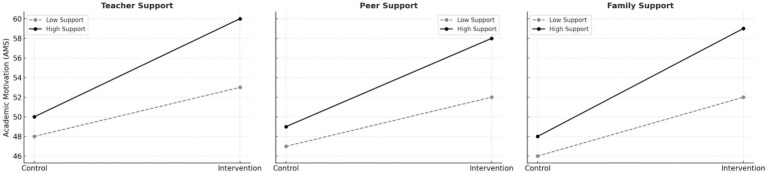
Interaction effects of teacher, peer, and family support on academic motivation.

The results in Table 6 and Figure 3 indicate that social support significantly moderated the relationship between the intervention and learning motivation. Teacher support (*β* = 1.20, *t* = 4.1, *p* < 0.001), family support (*β* = 1.05, *t* = 4.0, *p* < 0.001), and peer support (*β* = 0.95, *t* = 3.6, *p* < 0.01) each amplified the impact of the intervention. The three interaction curves shown in Figure 3 further reveal that students with stronger social support demonstrated substantial gains in learning motivation after the intervention, while those with limited support exhibited only modest improvement. This pattern suggests that social support functions as a decisive ecological factor shaping whether psychological interventions translate into sustained motivational change. The degree to which students’ relational networks can maintain emotional feedback and structural care appears central to the intervention’s success. Among these moderating influences, teacher support proved the most powerful, highlighting the distinctive role of educational relationships in linking psychological recovery with academic engagement for students managing chronic illness. Teachers act not only as educators but as social agents who help construct meaning and affirm self-worth. When relationships between teachers and students are built on trust and empathy, learners are more likely to channel the emotional and psychological gains of intervention into their studies. Relational trust, rather than instructional technique alone, appears to be the true catalyst for renewed motivation. Peer support, although somewhat weaker in effect, provided a vital sense of belonging that buffered emotional strain. For students living with chronic conditions, peer acceptance mitigated stigma and isolation, restoring a sense of connection and safety. Family support, in turn, grounded recovery in a broader social context: understanding and acceptance within the family created emotional continuity, extending the intervention’s benefits beyond campus life. At the same time, these positive effects reveal an underlying structural tension. The improvements in mental health and academic engagement still depend heavily on relational rather than institutional resources. The strength of social support as a moderator underscores the limited capacity of current educational and health systems to provide consistent structural support, leaving resilience to be sustained largely through personal networks of care. This reliance makes psychological recovery fragile: once those networks weaken, the gains achieved through intervention may quickly erode. Consequently, Table 6 and Figure 3 not only demonstrate the enhancing role of social support but also expose a deeper dilemma of educational equity. What appears as individual psychological progress is, in practice, embedded within unequal access to relational capital. The long-term task is not simply to expand supportive interactions but to convert relational care into institutional responsibility—to make social support a stable, systemic resource rather than a matter of personal goodwill. When psychological health and academic assistance become integral parts of the educational contract, recovery and learning can evolve into durable, equitable experiences rather than temporary responses to intervention.

**Table 6:** The moderating effect of social support.

Moderator	Group × Moderator β	*t*-value	Significance
Teacher support	1.2	4.1	***
Peer support	0.95	3.6	**
Family support	1.05	4	***

* indicate statistically significant differences at the 0.001 level (*p* < 0.05); ** indicate statistically significant differences at the 0.001 level (*p* < 0.01); *** indicate statistically significant differences at the 0.001 level (*p* < 0.001).

### 4.7 Heterogeneity analysis by disease type

The results in Table 7 indicate significant variation in intervention effectiveness across students with different chronic conditions. Among the three groups, students with diabetes showed the greatest improvement in learning motivation (*β* = 8.15, *t* = 7.8, *p* < 0.001), followed by those with epilepsy (*β* = 7.10, *t* = 6.1, *p* < 0.001), while the asthma group exhibited a smaller yet still significant effect (*β* = 6.00, *t* = 3.2, *p* < 0.01). These findings highlight the heterogeneous impact of the intervention across health conditions and reveal the deep interaction between psychological and physiological factors in educational adaptation. The substantial gains observed in the diabetes group suggest that the program activated stronger self-regulatory and goal-oriented behaviors within this population. Students managing diabetes often contend with ongoing challenges of disease control, dietary regulation, and self-monitoring, which cultivate a habitual sense of autonomy and behavioral discipline. When psychological support and educational resources were integrated systematically, these students appeared able to translate self-management skills into renewed academic motivation. In this sense, their improvement reflects the transferability of health management experience to learning behavior. However, the pronounced responsiveness of this group also points to a structural limitation of the intervention: psychological programs are more likely to succeed among individuals with “manageable” chronic conditions, whereas students experiencing greater physiological volatility—such as those with asthma or epilepsy—face more complex barriers shaped by both biological instability and social context. The relatively modest improvement among students with asthma suggests that the intervention may not have sufficiently addressed the unpredictability of their symptoms, which undermines a stable sense of safety and academic rhythm. For students with epilepsy, the gains were evident but primarily linked to enhanced emotional regulation and social acceptance rather than intrinsic motivational change. Because epilepsy is often associated with hidden stigma and social distancing, the intervention appears to have restored a sense of trust and self-recognition, which in turn supported re-engagement with learning activities. Taken together, these differentiated outcomes demonstrate that the effects of psychological intervention are not linear but mediated by the interaction of disease characteristics, social perception, and resource accessibility. The underlying tension lies in the contrast between the universal logic on which most educational interventions are designed and the deeply individualized and socially embedded nature of chronic illness experiences. This asymmetry produces structured disparities in how students benefit from support programs. Consequently, the effectiveness of educational–psychological intervention depends not only on methodological rigor but also on its capacity to engage with students’ lived experiences and social positions. Table 7 thus underscores a central challenge for interdisciplinary intervention: achieving a balance between differentiation and equity. The long-term significance of this finding lies in developing programs that do not rely on the controllability of specific diseases but can foster inclusive forms of psychological support and learning empowerment across diverse health contexts. The depth and sustainability of the integration between education and public health will ultimately depend on this capacity for structural inclusion.

**Table 7:** Heterogeneity analysis by disease type.

Disease type	Group β (AMS)	*t*-value	Significance
Diabetes	8.15	7.8	***
Asthma	6	3.2	**
Epilepsy	7.1	6.1	***

* indicate statistically significant differences at the 0.001 level (*p* < 0.05); ** indicate statistically significant differences at the 0.001 level (*p* < 0.01); *** indicate statistically significant differences at the 0.001 level (*p* < 0.001).

### 4.8 Feasibility and acceptability of the intervention

The results in Table 8 indicate that students generally showed high acceptance of the intervention. Among the three sources of support, teacher support had the highest mean score (*M* = 3.7), followed by family support (*M* = 3.6), and peer support was slightly lower (*M* = 3.4). While these findings suggest that the program was broadly welcomed in the educational setting, they also reveal distinct functional roles of each source of support across psychological and social dimensions. The strong level of teacher support highlights that the classroom remains the primary space for both psychological recovery and academic adjustment. Students often view teachers as mediators between institutional systems and emotional experience, and this trust helps anchor the intervention within everyday teaching. Teachers’ feedback, understanding, and empathy not only increased students’ engagement with the intervention but also contributed to rebuilding a sense of psychological safety within the school environment. Yet, the same high acceptance exposes a deeper structural issue—the concentration of “emotional labor” within educational systems. Although teachers’ involvement enhanced intervention outcomes, it also suggests that emotional care still depends heavily on personal effort rather than institutional provision. When instructional workloads rise or emotional fatigue sets in, such support networks can easily weaken. The relatively high rating of family support indicates that families played a stabilizing role in extending the benefits of the program beyond school. Acceptance and understanding at home allowed the effects of the intervention to persist, offering continuous emotional reinforcement. This outcome shows how the intervention reactivated the family’s supportive function, while also revealing inequalities linked to socioeconomic and cultural contexts. Students from resource-rich or education-oriented families more readily internalized the program’s principles, whereas stigma or communication barriers in some households limited the continuity of its impact. Peer support, by contrast, remained the lowest (*M* = 3.4), suggesting that peer relationships continue to be the most fragile aspect of psychological intervention. Although peer interaction is essential for belonging and emotional resonance, students with chronic illness often keep a degree of social distance—whether due to physical differences or self-protective habits—which constrains the formation of close peer ties. This pattern exposes a continuing tension between individual psychological improvement and collective social inclusion. Positive evaluations of the intervention therefore do not necessarily imply broader social acceptance, since the creation of a genuinely safe psychosocial environment remains hindered by cultural attitudes, group boundaries, and insufficient structural support. Taken together, Table 8 illustrates both the feasibility of the intervention and the persistent imbalance within educational and social systems. High levels of teacher and family support show that emotional resources are concentrated in a few relational domains, whereas weaker peer support underscores the need to rebuild networks of shared care. The long-term sustainability of such interventions depends on transforming care from an individual act into a collective educational culture—so that mental health becomes not a temporary remedy, but a core and institutionalized value of the school ecology.

**Table 8:** Feasibility and acceptability of the intervention.

Indicator	Mean	Explanation
Teacher support	3.7	Moderate to high
Peer support	3.4	Upper-medium
Family support	3.6	Moderate to high

## 5 Discussion

### 5.1 From intervention outcomes to mechanism explanation: the mediating role of mental health in the formation of academic motivation

The findings show that mental health served as a significant mediator between the interdisciplinary intervention and learning motivation, underscoring that psychological regulation operates as a central mechanism rather than a supplementary variable in educational practice. Following the intervention, students exhibited notable reductions in depression and anxiety (PHQ-9 and GAD-7 scores declined significantly) alongside improvements in self-esteem and perceived control. These outcomes indicate that the intervention alleviated emotional distress while helping students regain a sense of efficacy and control over their learning and future trajectories. In doing so, they achieved psychological balance amid the dual challenges of chronic illness and academic demand, redefining their learning goals through renewed emotional stability. This pattern accords with Self-Determination Theory, which holds that satisfying basic psychological needs strengthens intrinsic motivation. In this study, mental health acted as a psychological regulator that redirected learning from external compliance toward autonomous engagement by enhancing emotional regulation and reinforcing self-identity. Although the mediating effect was statistically significant, its magnitude was moderate (*a* × *b* = −0.14). Emotional recovery can activate learning motivation, yet its durability remains dependent on consistent institutional stability and continuous social support. Additional analyses revealed that the positive influence of mental health on learning engagement was stronger when students reported higher levels of social support, showing that psychological processes are always situated within relational and structural contexts. When emotional recovery occurs without sustained teacher support, family understanding, or institutional safeguards, its effects tend to diminish under external pressures. The boundaries of this mediating pathway reflect broader inequalities in the distribution of social resources. Thus, while the intervention improved psychological and academic outcomes, its success also highlights a form of social dependency—emotional recovery relies on resource allocation, and motivational persistence depends on recognition and inclusion within educational structures. Although mental health is often conceptualized as an individual trait, its expression in practice is shaped by processes of social reproduction. In the absence of structural care, psychological recovery functions more as temporary repair than as long-term empowerment. These findings call for a re-evaluation of current educational intervention models. When such programs become institutionalized as performance-driven initiatives, the focus on mental health risks becoming overly procedural, detached from the structural origins of psychological distress. To transform mental health into a sustainable source of intrinsic motivation, interventions must go beyond short-term emotional adjustment and instead reconfigure the affective order of education—linking teacher–student relationships, curriculum design, and support systems into a cohesive social process of motivation formation. Only under enduring institutional trust and genuine social care can the mediating chain of mental health evolve from transient recovery to enduring psychological and educational growth.

### 5.2 The amplifying effect of social support and institutional dependence: the social structural boundaries of intervention mechanisms

The results indicate that social support significantly moderated the relationship between the interdisciplinary intervention and learning motivation. Support from teachers, families, and peers all strengthened the intervention’s impact, with teacher support showing the strongest moderating effect (*β* = 1.20, *t* = 4.10, *p* < 0.001), followed by family (*β* = 1.05, *t* = 4.00, *p* < 0.001) and peer support (*β* = 0.95, *t* = 3.60, *p* < 0.01). These findings suggest that social support is not a background variable but a key condition shaping whether intervention benefits can take hold. Teacher support—expressed through feedback, encouragement, and modeling—provided students with both psychological safety and confidence. Family support created a stable emotional base that helped students sustain effort, while peer support strengthened belonging and social identity, giving learning motivation an affective and communal dimension. When these networks worked together, the psychological benefits of the intervention expanded, and emotional recovery was more likely to turn into sustained engagement in learning. At the same time, the large differences between students with high and low levels of support reveal the social dependency of these effects. Students who reported stronger support showed far greater motivational gains, indicating that psychological processes are not uniform but shaped by access to structural resources. Social support therefore carries a dual meaning: it can foster recovery and motivation, yet it can also reproduce existing inequalities. When teachers focus their attention on high achievers, when family resources follow socioeconomic hierarchies, and when school culture overlooks students with health conditions, support itself becomes embedded within unequal institutional arrangements. Under such conditions, differences in intervention outcomes reflect not only psychological variation but also the logic of resource distribution. As social support becomes a condition for success, intervention outcomes depend increasingly on structural advantage rather than individual effort. Psychological recovery, in this sense, turns into a socially conditioned process whose endurance depends on whether institutions can sustain emotional labor and resource flow. In performance-oriented systems that treat teachers’ emotional work as invisible and family care as private duty, mental health promotion remains structurally constrained. The amplifying power of social support is thus inherently fragile: it boosts outcomes but may also intensify disparities. To make social support a real mechanism of equity, education governance needs to shift from individualized remedies toward structural guarantees. This involves building institutional capacity for teachers’ socioemotional training, developing regularized school–family partnerships, and embedding peer-support networks within school life. When social support becomes a shared public resource rather than a form of private social capital, interventions can move beyond dependence on existing structures and help align psychological well-being with educational fairness in a lasting way.

### 5.3 Disease sensitivity and the stratified mechanism of educational inclusion: rethinking the equity of interdisciplinary intervention

The results show marked variation in the effects of the intervention across disease groups. Students with diabetes experienced the most substantial gains in mental health and learning motivation (mean increase = 9.4 points, *p* < 0.001), followed by those with epilepsy (mean increase = 8.7 points, *p* < 0.001). Improvements among students with asthma were smaller (mean increase = 5.8 points, *p* < 0.01). These differences go beyond physiology; they reflect the interwoven influences of health experience, social understanding, and educational context. Students managing diabetes or epilepsy often develop strong habits of self-regulation and consistent illness awareness. Because they are accustomed to monitoring their conditions and adjusting their behavior, they appear more capable of transforming the positive outcomes of psychological intervention into sustained academic focus. In contrast, students with asthma live with fluctuating symptoms that are easily affected by environmental conditions, which undermines the stability of both emotional balance and learning motivation. Asthma’s low visibility in school settings and its limited recognition among teachers and peers further contribute to a subtle, “invisible” marginalization that diminishes the intervention’s overall reach. Taken together, the findings point to a broader pattern: the success of intervention depends as much on social and institutional accommodation as on psychological response. Schools tend to design their support systems around health conditions that are predictable and easily managed, which unintentionally favors students whose illnesses fit a “typical” trajectory. What appears neutral in design can therefore reproduce inequality in practice—directing attention and resources toward groups that align with normative expectations while leaving others, particularly those with episodic or poorly understood conditions, at the margins. The supposed universality of intervention is thus conditional, grounded in implicit assumptions about what counts as a “normal” body or a legitimate learner. When these assumptions define fairness, inclusion risks becoming procedural rather than genuine. Equity, in this context, is not achieved through identical treatment but through institutional capacity to recognize and sustain difference. Educational and psychological interventions should be built with sensitivity to illness diversity, using flexible, adaptive, and differentiated approaches that reflect the varied emotional and cognitive demands associated with distinct health experiences. Schools can strengthen inclusion by aligning curriculum schedules with health rhythms, developing individualized assessment criteria, and providing teacher training that enhances health literacy. At the policy level, sustained networks of support are needed to distribute resources across diagnostic categories so that differences in health no longer translate into disparities in educational opportunity. When educational systems begin to acknowledge the full diversity of illness and revise their institutional logic accordingly, interventions can evolve beyond standardized frameworks and serve as lasting instruments of equity and human dignity.

## 6 Conclusion

This study investigated university students with chronic illnesses using an interdisciplinary psycho-educational intervention and explored how improvements in mental health, learning motivation, and social support interact within this process. The findings revealed notable post-intervention gains. Students’ mental health scores improved substantially, with mean reductions of 4.8 and 4.1 points in depression and anxiety (*p* < 0.001) and increases of 3.9 and 4.0 points in self-esteem and perceived control (*p* < 0.001). Learning motivation also rose markedly, by an average of 9.3 points (*p* < 0.001), suggesting that the intervention achieved significant benefits across both psychological and academic dimensions. The correlation between mental health and learning motivation (*r* = 0.52, *p* < 0.001) underscores the importance of emotional well-being in sustaining engagement with learning. In addition, the moderating role of social support highlights the essential contributions of teachers and families in translating psychological recovery into academic participation (*β* = 1.20 and 1.05, *p* < 0.001). At the same time, variations among disease groups—students with diabetes and epilepsy showing greater progress than those with asthma (*p* < 0.01)—point to the health-specific and structurally dependent nature of intervention outcomes. Such differences indicate that disparities in health experience, social understanding, and institutional provision continue to influence results unevenly. Overall, this study offers empirical evidence for the value of interdisciplinary intervention in improving both psychological recovery and academic adaptation while revealing persistent policy challenges concerning resource allocation and institutional inclusiveness in higher education. Theoretically, this research extends the dialog between educational and health psychology by showing that the interaction between psychological self-regulation and social support structures plays a central role in shaping academic motivation among students with chronic illness. Practically, the findings provide a framework for building integrated support systems in universities and emphasize that mental health promotion should be regarded not as an auxiliary service but as a key element of educational equity. From a critical perspective, although the intervention produced substantial gains, its long-term impact is likely to depend on institutional continuity and structural adaptability. Future studies should therefore investigate the durability of intervention effects, the responsiveness of institutional policies, and the fairness of resource distribution among diverse health populations. Advancing in these directions may help higher education evolve from individualized psychological assistance toward systemic forms of structural support, enabling mental health to serve as a shared foundation for educational inclusion and sustainable academic growth.

## 7 Limitations

While this study provides systematic evidence for the effectiveness of interdisciplinary intervention in improving the mental health and learning motivation of university students with chronic illnesses, several limitations merit consideration. The sample was drawn from a small number of universities with limited regional and disciplinary diversity. Differences in institutional culture and health-care provision may have unintentionally influenced the consistency of intervention outcomes, constraining the generalizability of the findings. The measures relied primarily on self-reported data, which are vulnerable to subjective bias and social-desirability effects; improvements in psychological well-being and motivation may therefore partly reflect short-term cognitive shifts rather than enduring behavioral change. Moreover, the evaluation focused mainly on short-term outcomes and lacked longitudinal follow-up, making it difficult to assess whether psychological and behavioral improvements were sustained or translated into long-term academic performance. Although disease type was included as a variable, the analysis did not fully explore how illness trajectories, symptom variability, and medical support interacted with the intervention, leaving the mechanisms of health sensitivity only partially understood. Finally, the study did not differentiate between structural and emotional components of social support or consider how institutional policy environments shape the sustainability of intervention effects, limiting deeper insight into issues of educational equity and resource distribution. Taken together, this research provides an empirical foundation for understanding the psychological health and academic adjustment of students with chronic illness, but its conclusions should be tested across broader populations, cultural settings, and institutional contexts. Future studies could integrate longitudinal designs, mixed methods, and policy-level analyses to examine the persistence of intervention effects and the structural boundaries that condition them. Advancing along these lines would promote closer integration of mental-health initiatives and inclusive education within higher-education systems, helping to build more equitable and sustainable forms of psychosocial support.

## Data Availability

The raw data supporting the conclusions of this article will be made available by the authors, without undue reservation.
